# The Selective Serotonin 2A Receptor Antagonist Sarpogrelate Prevents Cardiac Hypertrophy and Systolic Dysfunction via Inhibition of the ERK1/2–GATA4 Signaling Pathway

**DOI:** 10.3390/ph14121268

**Published:** 2021-12-05

**Authors:** Kana Shimizu, Yoichi Sunagawa, Masafumi Funamoto, Hiroki Honda, Yasufumi Katanasaka, Noriyuki Murai, Yuto Kawase, Yuta Hirako, Takahiro Katagiri, Harumi Yabe, Satoshi Shimizu, Nurmila Sari, Hiromichi Wada, Koji Hasegawa, Tatsuya Morimoto

**Affiliations:** 1 Division of Molecular Medicine, School of Pharmaceutical Sciences, University of Shizuoka, Shizuoka 422-8526, Japan; s18804@u-shizuoka-ken.ac.jp (K.S.); y.sunagawa@u-shizuoka-ken.ac.jp (Y.S.); funamoto@u-shizuoka-ken.ac.jp (M.F.); bm0320hiro@yahoo.co.jp (H.H.); katana@u-shizuoka-ken.ac.jp (Y.K.); m.noriyuki.22@gmail.com (N.M.); m17032@u-shizuoka-ken.ac.jp (Y.K.); m18171@u-shizuoka-ken.ac.jp (Y.H.); m17028@u-shizuoka-ken.ac.jp (T.K.); m16112@u-shizuoka-ken.ac.jp (H.Y.); s18410@u-shizuoka-ken.ac.jp (S.S.); nurmilasari@gmail.com (N.S.); koj@kuhp.kyoto-u.ac.jp (K.H.); 2 National Hospital Organization Kyoto Medical Center, Division of Translational Research, Kyoto 612-8555, Japan; hwada@kuhp.kyoto-u.ac.jp; 3 Shizuoka General Hospital, Shizuoka 420-8527, Japan

**Keywords:** sarpogrelate, 5-HT_2A_ receptor, drug repositioning, ERK1/2–GATA4 pathway, heart failure

## Abstract

Drug repositioning has recently emerged as a strategy for developing new treatments at low cost. In this study, we used a library of approved drugs to screen for compounds that suppress cardiomyocyte hypertrophy. We identified the antiplatelet drug sarpogrelate, a selective serotonin-2A (5-HT_2A_) receptor antagonist, and investigated the drug’s anti-hypertrophic effect in cultured cardiomyocytes and its effect on heart failure in vivo. Primary cultured cardiomyocytes pretreated with sarpogrelate were stimulated with angiotensin II, endothelin-1, or phenylephrine. Immunofluorescence staining showed that sarpogrelate suppressed the cardiomyocyte hypertrophy induced by each of the stimuli. Western blotting analysis revealed that 5-HT_2A_ receptor level was not changed by phenylephrine, and that sarpogrelate suppressed phenylephrine-induced phosphorylation of ERK1/2 and GATA4. C57BL/6J male mice were subjected to transverse aortic constriction (TAC) surgery followed by daily oral administration of sarpogrelate for 8 weeks. Echocardiography showed that 5 mg/kg of sarpogrelate suppressed TAC-induced cardiac hypertrophy and systolic dysfunction. Western blotting revealed that sarpogrelate suppressed TAC-induced phosphorylation of ERK1/2 and GATA4. These results indicate that sarpogrelate suppresses the development of heart failure and that it does so at least in part by inhibiting the ERK1/2–GATA4 signaling pathway.

## 1. Introduction

Heart failure is a serious public health problem, with an estimated 64 million cases under treatment globally each year [1,2]. As the number of people suffering from the disease is expected to continue increasing over the coming decades, heart failure is one of the most significant health problems worldwide [3,4]. Neurohormonal antagonists, including β blockers, and renin-angiotensin system inhibitors are the established standards for heart failure therapy, based on clinical studies demonstrating that these drugs suppress cardiac remodeling and improve prognosis [5,6]. However, as prognosis remains poor even with the use of these drugs, new heart failure therapies are urgently needed [5,6].

Drug discovery and development are high-risk ventures that are expensive, labor intensive, and time consuming. The Pharmaceutical Research and Manufacturers of America has reported that the development of a new drug takes an average of US $2.6 billion and from 10 to 15 years from project initiation to the United States Food and Drug Administration’s approval [7]. For this reason, more efficient drug discovery strategies are required. One strategy that has recently been garnering attention is drug repositioning, in which new treatments are developed at low cost and low health risk by investigating the efficacy of previously approved drugs for additional diseases [8]. The major advantage of this approach is that the pharmacokinetic and safety profiles of such drugs have already been established [9]. It has been reported that drug repositioning accounts for approximately 30% of recent drug approvals [10,11].

Sarpogrelate is a selective serotonin-2A (5-HT_2A_) receptor antagonist that inhibits 5-HT-induced physiological effects, including platelet aggregation, vasoconstriction, and the proliferation of vascular smooth muscle cells mediated by 5-HT_2A_ receptors [12]. It has been used as a treatment for peripheral arterial occlusive disease in Asia [13,14]. It has also been reported to have therapeutic efficacy for thrombosis, coronary artery disease, and atherosclerosis [15], and to have protective effects against damage to retinal neurons, against endothelial and renal dysfunction, and against oxidative stress induced by high glucose in diabetes model animals [15,16,17,18]. These findings suggest that sarpogrelate may also be effective for the treatment of heart disease.

In this study, we focused on a drug repositioning strategy for heart failure therapy using a library of approved drugs to screen for compounds that suppress phenylephrine (PE)-induced hypertrophy in primary cultured cardiomyocytes. Sarpogrelate was identified as being capable of suppressing PE-induced cardiomyocyte hypertrophy. As it was unclear whether sarpogrelate suppresses PE-induced cardiomyocyte hypertrophy in a 5-HT_2A_-dependent or -independent manner, we investigated the effect of sarpogrelate both on cardiomyocyte hypertrophy and on the development of heart failure in mice. We found that sarpogrelate suppressed PE-induced cardiomyocyte hypertrophy in a 5-HT_2A_ receptor-independent manner by inhibiting the ERK1/2–GATA4 signaling pathway and by suppressing pressure overload-induced cardiac hypertrophy and systolic dysfunction in vivo.

## 2. Results

### 2.1. Sarpogrelate Suppressed Cardiomyocyte Hypertrophy Induced by Various Hypertrophic Stimuli

**Figure 1:**
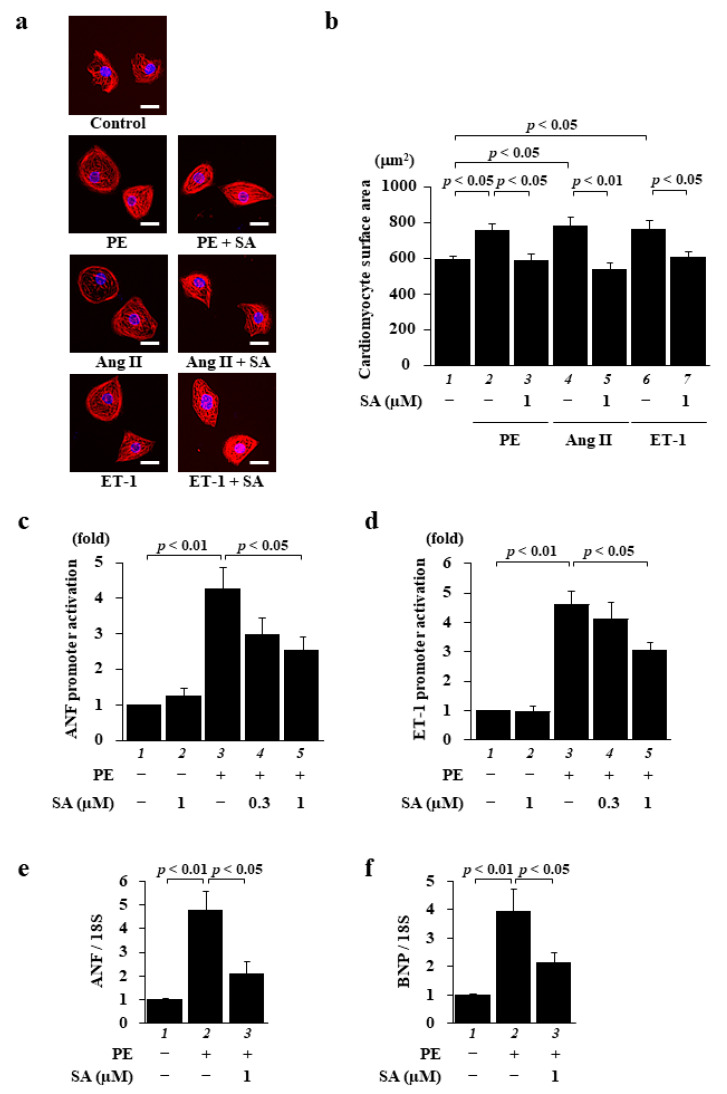
Sarpogrelate suppressed cardiomyocyte hypertrophy induced by a variety of hypertrophic stimuli. Primary cultured cardiomyocytes were treated with 0.3 or 1 μM sarpogrelate (SA) and then stimulated with 30 μM phenylephrine (PE), 0.1 µM angiotensin II (Ang II), or 0.1 µM endothelin 1 (ET-1) for 48 h. (**a**) Immunofluorescence staining was performed using anti-MHC antibodies and Alexa Fluor 555-conjugated anti-mouse IgG. Scale bar: 20 μm. (**b**) Cell surface area was measured using NHI ImageJ software. All data are presented as the mean ± SEM of four individual experiments. (**c**,**d**) Cardiomyocytes were harvested 48 h after stimuli and a luciferase reporter assay was performed for ANF (**c**) and ET-1 (**d**) promoters. Data are presented as the mean ± SEM of five individual experiments. (**e**,**f**) The mRNA levels of hypertrophy-related gene transcriptions of ANF (**e**) and BNP (**f**) at 48 h after stimulation were examined by quantitative PCR. Data are presented as the mean ± SEM of three individual experiments.

First, to investigate the effect of sarpogrelate on cardiomyocyte hypertrophy induced by various types of hypertrophic stimuli, primary cultured cardiomyocytes were pretreated with 1 µM sarpogrelate, and then cell hypertrophy was induced separately with the hypertrophic stimuli PE, angiotensin II (Ang II), and endothelin1 (ET-1) for 48 h. Surprisingly, 1 µM sarpogrelate suppressed the cardiomyocyte hypertrophy induced by each stimulant (Figure 1a,b). Next, to investigate the effect of sarpogrelate on the PE-induced promoter activity of hypertrophic response genes in cultured cardiomyocytes, a luciferase reporter assay using the atrial natriuretic factor (ANF)-luc and ET-1-luc reporter genes was performed. The results showed that sarpogrelate suppressed PE-induced ANF- (Figure 1c) and ET-1-luciferase (Figure 1d) expression. Quantitative PCR analysis showed that sarpogrelate suppressed the PE-induced hypertrophy-related gene transcription of ANF and brain natriuretic peptide (BNP) (Figure 1e,f). These results suggest that sarpogrelate directly suppressed hypertrophic response in cultured cardiomyocytes.

### 2.2. Sarpogrelate Inhibited the ERK1/2–GATA4 Signaling Pathway in Cardiomyocytes

**Figure 2:**
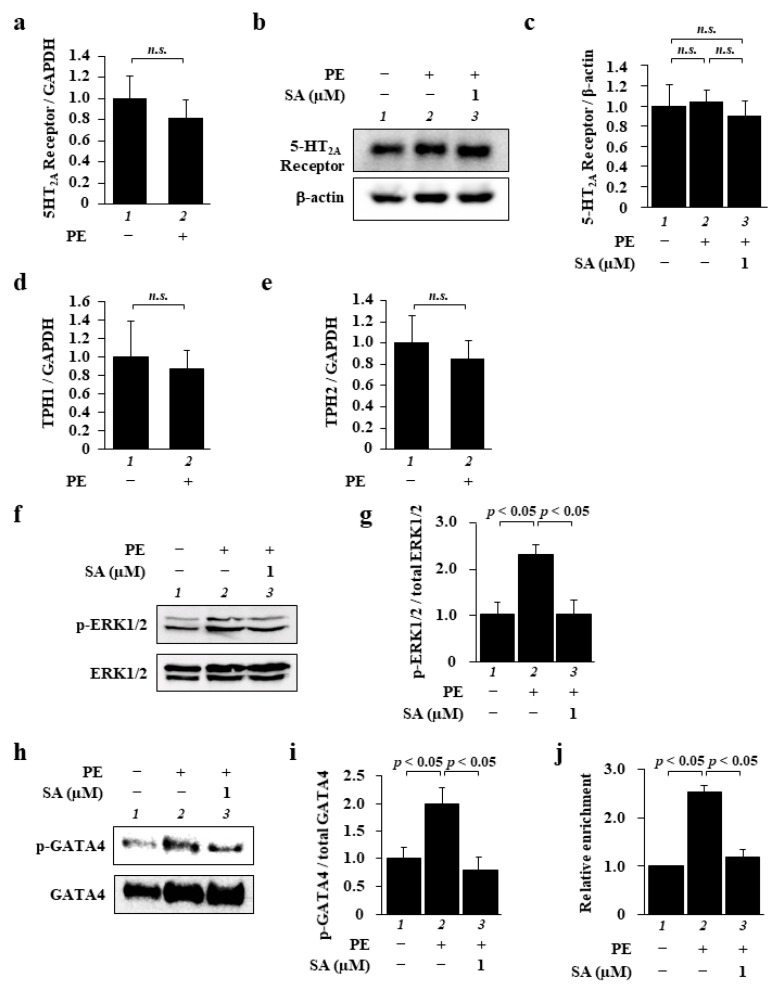
Sarpogrelate inhibited the ERK1/2–GATA4 signaling pathway in cardiomyocytes. (**a**) The mRNA levels of the 5-HT_2A_ receptor were examined at 48 h after PE stimulation. Quantitative PCR data are presented as the mean ± SEM of three individual experiments. (**b**,**c**) WCL was extracted from cardiomyocytes at 48 h after PE stimulation. Representative Western blotting image (**b**) and quantified 5-HT_2A_ receptor levels (**c**). Quantification is presented as the mean ± SEM of three individual experiments; n.s., no significance. (**d**,**e**) Quantitative PCR was performed for TPH1 (**d**), TPH2 (**e**), and 18S. Data are presented as the mean ± SEM of three individual experiments; n.s., no significance. (**f**) WCL was extracted from cardiomyocytes at 10 min after PE stimulation and then subjected to Western blotting using anti-phospho-p44/42 MAPK (ERK1/2) (Thr202/Tyr204) antibody and anti-p44/42 MAPK (ERK1/2) antibody. (**g**) Levels of phosphorylated ERK1/2 and total ERK1/2 were quantified. Data are presented as the mean ± SEM of three individual experiments. (**h**) The NE fraction was isolated from the cells at 1 h after PE stimulation and then subjected to Western blotting using anti-GATA4 (phospho S105) antibody and GATA4 antibody. (**i**) Levels of phosphorylated GATA4 and total GATA4 were quantified. Data are presented as the mean ± SEM of three individual experiments. (**j**) A ChIP assay was performed using anti-GATA4 antibody, and quantitative PCR was performed for ANF and 18S. Data are presented as the mean ± SEM of three individual experiments.

Next, we investigated how sarpogrelate suppresses PE-induced cardiomyocyte hypertrophy. As it is possible both that PE induces increases in 5-HT_2A_ receptor expression and/or 5-HT levels and that sarpogrelate suppresses cardiomyocyte hypertrophy by inhibiting the 5-HT_2A_ receptor, we tested whether PE induces changes both in the expression of the receptor and in 5-HT synthesis. First, to investigate whether phenylephrine increases the expression level of the 5-HT_2A_ receptor, we examined the mRNA and protein levels of the receptor. Quantitative PCR analysis and Western blotting demonstrated that PE did not change the mRNA (Figure 2a) or protein expression (Figure 2b,c) of the receptor. Next, as it has been reported that 5-HT is synthesized in the heart by the enzymes tryptophan hydroxylase-1 (TPH1) and -2 (TPH2) [19], we investigated the mRNA levels of these enzymes. The results of quantitative PCR analysis showed that these mRNA levels were not changed by PE (Figure 2d,e), indicating that 5-HT synthesis was not affected by PE. We also confirmed both that PE induced cardiomyocyte hypertrophy under conditions of 5-HT_2A_ receptor knockdown, and that the suppressive effect of sarpogrelate on PE-induced cardiomyocyte hypertrophy was not affected by this knockdown (Supplementary Figure S1). These findings suggest that sarpogrelate suppresses cardiomyocyte hypertrophy without increasing 5-HT_2A_ receptor expression or 5-HT levels.

Next, to explore the mechanism by which sarpogrelate suppresses cardiomyocyte hypertrophy, we focused on the ERK1/2–GATA4 signaling pathway, which is one of the main signaling pathways involved in cardiomyocyte hypertrophy. The results of Western blotting showed that sarpogrelate suppressed PE-induced ERK1/2 phosphorylation (Figure 2f,g). Similarly, sarpogrelate suppressed GATA4 phosphorylation (Figure 2h,i). The total expression of ERK1/2 and GATA4 was not changed by PE or sarpogrelate (Figure 2f,h). To further investigate the downstream effects of sarpogrelate on this signaling pathway, we investigated the amount of GATA4 binding to the ANF promoter, one of the main target promoters of GATA4. The results of a chromatin immunoprecipitation (ChIP) assay using anti-GATA4 antibody revealed that sarpogrelate suppressed the PE-induced enrichment of GATA4 in the ANF promoter region (Figure 2j). These results suggest that sarpogrelate suppresses cardiomyocyte hypertrophy by inhibiting the ERK1/2–GATA4 signaling pathway in a 5-HT_2A_ receptor-independent manner.

### 2.3. Sarpogrelate Suppressed Transverse Aortic Constriction (TAC)-Induced Cardiac Hypertrophy and Systolic Dysfunction

**Figure 3:**
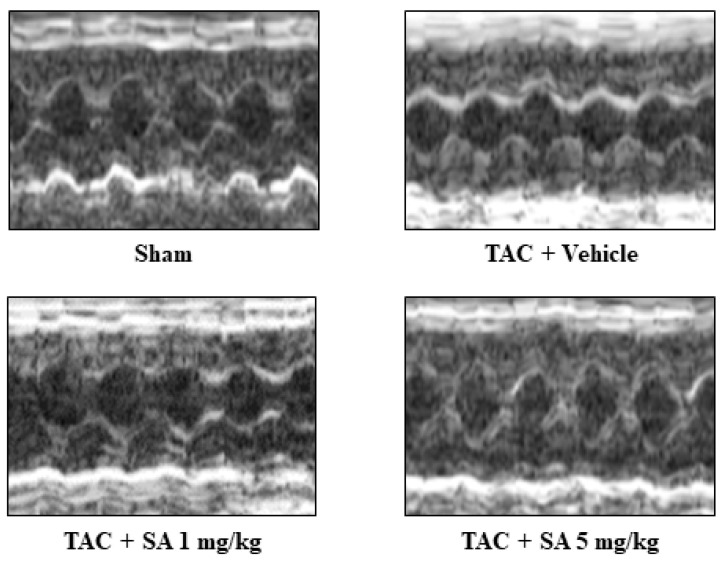
Sarpogrelate suppressed TAC-induced cardiac hypertrophy and systolic dysfunction in vivo. Eight weeks after TAC surgery, cardiac hypertrophy and cardiac function were assessed by echocardiography. Representative images of echocardiography from sarpogrelate- and vehicle-treated TAC and sham are shown.

To examine whether sarpogrelate suppresses the development of heart failure, C57BL/6J male mice were subjected to TAC surgery, and then these mice were randomly assigned to daily oral administration of 1 or 5 mg/kg sarpogrelate. The results of echocardiography at eight weeks after surgery revealed that 5 mg/kg sarpogrelate suppressed TAC-induced increases in left ventricular posterior wall dimensions (LVPWd) and LV mass index, the standard parameters of cardiac hypertrophy (Figure 3 and Table 1). Five mg/kg sarpogrelate also suppressed TAC-induced decreases in fractional shortening (FS), the standard parameters of cardiac function (Figure 3 and Table 1). After the assessment of cardiac function by echocardiography, the hearts were isolated, and the ratio of heart weight to tibia length (HW/TL) was calculated for all of the mice. The results showed that HW/TL ratio was increased by TAC surgery, and that this change was suppressed by 5 mg/kg sarpogrelate (Table 1).

### 2.4. Sarpogrelate Suppressed TAC-Induced Cardiac Hypertrophy and Fibrosis

**Figure 4:**
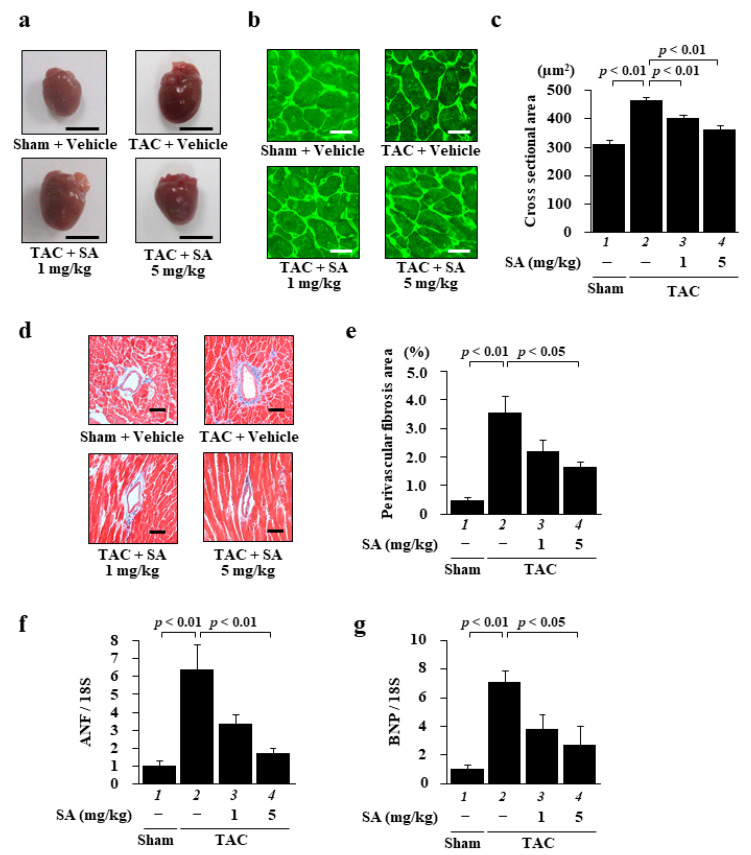
Sarpogrelate suppressed TAC-induced cardiac hypertrophy and fibrosis. (**a**) Representative image of isolated heart from sham and TAC mice at 8 weeks after surgery. Scale bar: 5 mm. (**b**) Representative images of WGA-stained sections of LV myocardium from sham and TAC mice. Magnification: ×400. Scale bar: 20 μm. (**c**) Cardiomyocyte cross-sectional area was measured for 50 cells from five to seven mice from each group. (**d**) Representative photographs of the MT-stained perivascular fibrosis area of the LV myocardium of sham and TAC mice. Magnification: ×200. Scale bar: 50 μm. (**e**) The area of perivascular fibrosis in the LV was measured for at least three intramyocardial coronary arteries in each animal. Data are presented as the mean ± SEM of five to six individual experiments. (**f**,**g**) Quantitative PCR analyses were performed for ANF (**f**) and BNP (**g**). Data are presented as the mean ± SEM of five individual experiments.

To characterize the effect of sarpogrelate on heart failure, we quantified heart cross-sectional area and perivascular fibrosis area by staining the tissue with wheat germ agglutinin (WGA) and Masson trichrome (MT) staining, respectively. WGA staining showed that sarpogrelate suppressed a TAC-induced increase in the cross-sectional area of the left ventricle (LV) (Figure 4b,c), and MT staining showed that sarpogrelate also suppressed TAC-induced perivascular fibrosis (Figure 4d,e). Subsequently, the mRNA levels of ANF and BNP in the LV were investigated by quantitative PCR analysis. The results showed that TAC-induced increases in the mRNA expression levels of ANF (Figure 4f) and BNP (Figure 4g) were suppressed by sarpogrelate.

### 2.5. Sarpogrelate Suppressed TAC-Induced Phosphorylation of ERK1/2 and GATA4

**Figure 5:**
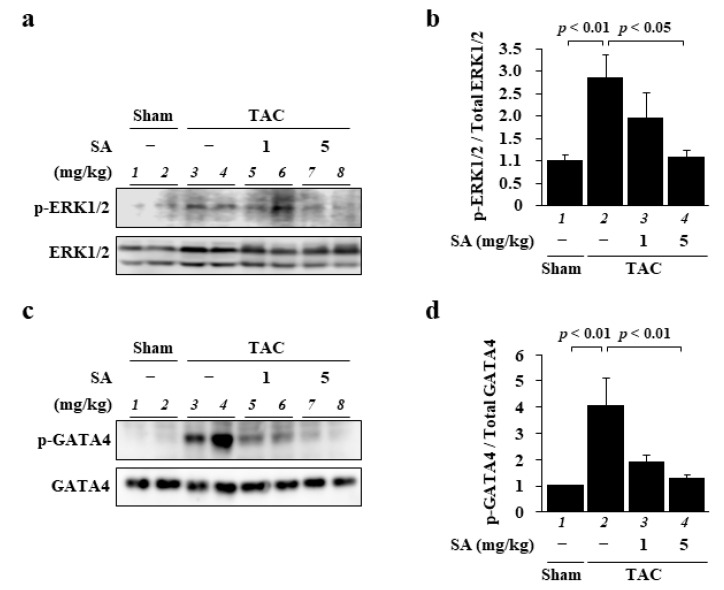
Sarpogrelate suppressed TAC-induced phosphorylation of ERK and GATA4. (**a**,**b**) WCL from mouse heart was subjected to Western blotting to assess the levels of phosphorylated ERK1/2 and total ERK. Representative Western blotting images are shown in (**a**) and quantified levels of phosphorylated and total ERK1/2 in (**b**). (**c**,**d**) NE fractions from mouse heart were subjected to Western blotting to assess phosphorylated GATA4 and total GATA4. Representative images of Western blotting are shown in (**c**) and quantified phosphorylated and total GATA4 levels in (**d**). All data are presented as the mean ± SEM of four individual experiments.

To investigate the potential role of sarpogrelate in the development of heart failure, its effect on TAC-induced activation of the ERK1/2–GATA4 signaling pathway was assessed with Western blotting. The results showed that the phosphorylation level of ERK1/2 was increased by TAC surgery, and also that sarpogrelate suppressed this increase (Figure 5a,b). Sarpogrelate also suppressed TAC-induced GATA4 phosphorylation (Figure 5c,d). The total expression of ERK1/2 and GATA4 was not changed by TAC surgery or sarpogrelate (Figure 5a,c). These results suggest that the protective effect of sarpogrelate against the development of heart failure is dependent on, at least in part, the inhibition of the ERK1/2–GATA4 signaling pathway.

## 3. Discussion

This study demonstrates that the antiplatelet drug sarpogrelate, a selective 5-HT_2A_ receptor antagonist, suppresses cardiomyocyte hypertrophy induced by various hypertrophic stimuli such as PE, Ang II and ET-1. It also suggests that sarpogrelate suppresses PE-induced cardiomyocyte hypertrophy not by mediating 5-HT/5-HT_2A_ receptors but by inhibiting the ERK1/2–GATA4 signaling pathway. It also reveals that sarpogrelate suppresses the development of heart failure via inhibition of the ERK1/2–GATA4 pathway in a heart failure mouse model. These results suggest that sarpogrelate may be effective not only as an antiplatelet drug but also as a treatment for heart failure due to its suppression of cardiomyocyte hypertrophy.

This study suggests that sarpogrelate suppresses PE-induced cardiomyocyte hypertrophy without mediation through the 5-HT_2A_ receptor. The monoamine neurotransmitter 5-HT regulates a wide range of physiological functions [20]. Although it is well established that 5-HT is synthesized by the enzyme TPH in enterochromaffin cells in the gastrointestinal tract and then released into the blood [21], TPH expression has also been reported to occur in hamster heart [19]. This indicates the possibility that 5-HT acts on the heart in both autocrine and paracrine fashion. Moreover, in cultured cardiomyocytes, it has been reported that 5-HT induced cardiomyocyte hypertrophy via the 5-HT_2A_ receptor [22,23]. In the present study, the expression levels of TPH1 and TPH2 were not changed by PE or sarpogrelate. This indicates that 5-HT synthesis was not affected by PE, and that 5-HT is not involved in PE-induced cardiomyocyte hypertrophy. We have also demonstrated that 5-HT_2A_ receptor expression was not changed by PE or sarpogrelate, and that knockdown of the 5-HT_2A_ receptor did not affect the anti-hypertrophy effect of sarpogrelate on PE-induced cardiomyocyte hypertrophy. These results suggest that sarpogrelate suppresses cardiomyocyte hypertrophy without the mediation of the 5-HT/5-HT_2A_ receptor.

Over 200 kinds of GPCRs have been found in the heart. Among them, the signaling pathways activated by G_q/11_ protein play a particularly important role in cardiac hypertrophy [24,25]. The mitogen-activated protein kinase (MAPK) signaling pathway, which is downstream from the G_q/11_ protein activated by PE, Ang II, and ET-1, is an important pathway in cardiomyocyte hypertrophy and the development of heart failure [26,27]. The MAPK pathway consists of at least three cascades in mammalian cells, namely ERK1/2, p38, and c-Jun N-terminal kinases (JNKs). Each cascade typically consists of at least three types of sequential protein kinases, including MAP kinase (MAPK), MAPK kinase (MAPKK), and MAPK kinase kinase (MAPKKK) [28,29]. Among the MAPKs, ERK1/2 in particular has been reported to be an important factor in the transcription of hypertrophic response genes [30,31]. In the present study, we found that sarpogrelate suppressed the phosphorylation of both ERK1/2 and GATA4 induced by PE in cultured cardiomyocytes. It has been reported that ERK1/2 phosphorylation is induced by the stimulation of Ang II and ET-1 as well as PE [32,33]. This suggests that sarpogrelate also suppresses Ang II- and ET-1-induced phosphorylation of ERK1/2. Proteins that regulate ERK1/2 phosphorylation include MAPKK MEK1 and phosphatases such as protein phosphatase 2A (PP2A) and the largest family of MAPK-selective phosphatases known as dual-specificity phosphatases (DUSPs) [31,34]. The overexpression of these MAPK-selective phosphatases suppresses cardiac hypertrophy by negatively modulating MAPKs mediated by dephosphorylation activity [34]. It is possible that sarpogrelate suppresses ERK1/2 phosphorylation by increasing the expression levels of these phosphatases.

Nuclear factor of activated T-cells (NFATc) is a representative transcriptional factor which acts together with GATA4 in cardiomyocyte hypertrophy. It has been reported that transcriptional cross-talk between the MEK1-ERK1/2 and calcineurin-NFATc signaling pathways is required for cardiac hypertrophy [35]. It also has been reported that overexpression both of NFATc and GATA4 in cardiomyocytes causes synergistic activation of the BNP gene promoter [36,37]. Thus, it is possible that sarpogrelate suppresses the PE-induced transcriptional activation of NFATc.

Cardiac remodeling accompanied by morphological and structural changes occurs in the process of heart failure induced by chronic stresses such as hypertension [38]. At the beginning of this process, neurohormonal factors (noradrenaline, Ang II, ET-1, etc.) are secreted and activate the G protein-coupled receptors (GPCRs) found on cell membranes [39]. Consequently, various signaling pathways are activated, eventually activating the expression of hypertrophic response gene transcription [40]. In the present study, sarpogrelate suppressed cultured cardiomyocyte hypertrophy induced by all of the stimulants that are recognized as representative neurohormonal factors related to heart failure. We have also demonstrated that sarpogrelate suppressed TAC-induced cardiac hypertrophy and systolic dysfunction. Thus, it is highly likely that sarpogrelate effectively suppresses pathological cardiac hypertrophy by directly suppressing the cardiomyocyte hypertrophy induced by various neurohormonal factors in mice.

There are several reports on the contribution of 5-HT and its receptor to the onset and progression of heart failure. In chronic heart failure patients, blood levels of 5-HT are correlated with the progression of the disease [41]. It has been reported that the selective 5-HT_2A_ receptor antagonist M100907 suppresses pressure overload-induced cardiac hypertrophy by inhibiting the CaMKII/HDAC4 pathway [42]. As described above, 5-HT expression is increased in heart failure, and 5-HT directly induces cardiomyocyte hypertrophy via the 5-HT_2A_ receptor [22,23,41]. These findings indicate that M100907 suppresses the CaMKII/HDAC4 pathway, which is most likely downstream of the 5-HT_2A_ receptor, resulting in the prevention of cardiac hypertrophy in mice. In addition to elucidating the mechanism by which sarpogrelate suppresses cardiomyocyte hypertrophy induced by PE, Ang II, and ET-1 in a 5-HT_2A_ receptor-independent manner, these findings also suggest that sarpogrelate suppresses 5-HT-induced cardiomyocyte hypertrophy via 5-HT_2A_ receptor blockade, in which case the mechanism may also be involved in the suppression of the CaMKII/HDAC4 pathway. Therefore, the present study suggests that sarpogrelate suppresses TAC-induced cardiac hypertrophy through both 5-HT_2A_ receptor-independent and dependent mechanisms. Moreover, while mRNA and protein expression of the 5-HT_2A_ receptor were not changed by PE stimulation in the present study, it has been reported that protein expression of the 5-HT_2A_ receptor was increased in TAC mice at 1 month after surgery [42]. It has also been reported that 5-HT_2A_ receptor expression is increased by 5-HT stimulation in H9c2 cells and fetal myoblasts [23,43]. As the heart is made up of fibroblasts, myoblasts, and endothelial cells in addition to cardiomyocytes [44], it is likely that increased 5-HT expression in heart failure also induces the upregulation of the 5-HT_2A_ receptors in these cells, thereby increasing 5-HT_2A_ receptor expression in TAC mice. This suggests that 5-HT_2A_ receptor expression is not affected by PE because 5-HT synthesis is not facilitated by PE in cultured cardiomyocytes.

The present study also demonstrates that sarpogrelate suppressed TAC-induced phosphorylation of ERK1/2 and GATA4 in mice. There are several reports that describe the role of MEK-ERK1/2 as a main MAPK pathway in cardiac hypertrophy and heart failure. Many studies have reported that a variety of compounds, including the MEK inhibitor PD98059, both inhibit ERK1/2 phosphorylation and suppress cardiac hypertrophy [31,45]. However, it has also been reported that the deletion of cardiac-specific ERK1/2 causes eccentric hypertrophy and subsequently reduced cardiac function 2 weeks after Ang II/PE infusion in mice [46]. In contrast, the overexpression of MEK1, which is an upstream kinase of ERK1/2, also induced cardiac hypertrophy through the phosphorylation of ERK1/2 in mice [47]. These findings make it clear that ERK1/2 plays a complex role in the progress of cardiac hypertrophy and suggest that the complete abolishment of ERK1/2 induces the development of cardiac hypertrophy. In the present study, sarpogrelate suppressed TAC-induced phosphorylation of ERK1/2, but it did not completely suppress eccentric hypertrophy, suggesting the possibility that sarpogrelate does not inhibit the activation of MEK1 in cardiac hypertrophy. Further study is needed to determine whether the inhibition of ERK1/2, including the inhibition caused by sarpogrelate, may be effective for heart failure therapy.

Many studies have shown increased platelet activation in heart failure [48]. Indeed, the Antithrombotic Therapy Trialists’ Collaboration meta-analysis reported that anti-platelet agents are effective for patients at risk of ischemic events [48]. Sarpogrelate is used as a treatment for peripheral vascular disease by suppressing 5-HT-induced platelet aggregation and vasoconstriction [49]. It has been reported that sarpogrelate suppressed systolic dysfunction by attenuating the remodeling of subcellular organelles such as the sarcoplasmic reticulum and myofibrils, and by exerting vasodilatory effects in a rat model of congestive heart failure in post-myocardial infarction [50]. This indicates that sarpogrelate effectively suppressed cardiac hypertrophy not only by direct suppression of cardiomyocyte hypertrophy, but also through its cardio-protective effect as an anti-platelet agent. While we have not studied the effect of sarpogrelate on hearts that already have decreased systolic function, it has been reported that 6 months of sarpogrelate-based triple antiplatelet therapy in patients undergoing primary percutaneous coronary intervention for ST-elevation myocardial infarction improved LV systolic dysfunction more effectively than dual antiplatelet therapy without sarpogrelate [51]. These findings suggest that sarpogrelate may also be effective in hearts with reduced cardiac function.

## 4. Materials and Methods

### 4.1. Materials

Sarpogrelate hydrochloride was provided by Mitsubishi Tanabe Pharma Corporation (Osaka, Japan). PE and ET-1 were purchased from Fujifilm Wako Pure Chemical Industries (Osaka, Japan), and Ang II was purchased from Bachem (Torrance, CA, USA). These compounds were dissolved in Milli-Q water and stored at −20 °C.

### 4.2. Plasmid Constructs

The pANF-luc and pET-1-luc plasmids consisted of firefly luciferase cDNA driven by a 170 bp ANF promoter sequence from −135 to +35 and a 213 bp ET-1 promoter sequence from −213 to −1. Both promoter sequences contained a single GATA element.

### 4.3. Animal Experiments

Male Sprague-Dawley rats were purchased from Japan SLC Inc. (Shizuoka, Japan). C57BL/6j male mice were purchased from CREA Japan Inc. (Tokyo, Japan). All animal experiments complied with the guidelines on animal experiments of the University of Shizuoka (Shizuoka, Japan) and Kyoto Medical Center (Kyoto, Japan) and were performed in accordance with protocols approved by the ethics committees of the two institutions (approval numbers 156161 and 27-26-2, respectively).

### 4.4. Cell Culture

Neonatal rat ventricular myocytes were isolated from 1-2-day-old Sprague-Dawley rats as described previously [52,53]. The cells were pretreated with 1 µM sarpogrelate for two hours and then treated separately with each stimulus (PE, 30 µM; Ang II, 0.1 µM; ET-1, 0.1 µM). To assess hypertrophy, cells were incubated with the stimuli for 48 h.

### 4.5. Sample Preparation

Whole cell lysate (WCL) and nuclear extract (NE) were isolated as described previously [54,55]. In brief, the cells were harvested, and WCL was prepared on ice with cell lysis buffer (50 mM Tris-HCl pH 8.0, 150 mM NaCl, 2% Nonidet P40, 0.2 mM ethylenediaminetetraacetic acid) for 5 min. NE was extracted from the cells using the Dignam method [56]. The samples were subsequently resolved by Western blotting.

### 4.6. Immunofluorescence Staining

Immunofluorescence staining was performed as described previously [57,58]. Forty-eight hours after stimulation with PE, Ang II and ET-1, the cardiomyocyte cytoplasm was stained with anti-myosin heavy chain (MHC) antibody (Leica Biosystems, Nussloch, Germany) and Alexa Fluor 555-conjugated anti-mouse IgG (Invitrogen, Carlsbad, CA, USA), and the nuclei were stained with Hoechst 33258 (Dojinjo, Kumamoto, Japan). Fifty cardiomyocytes were randomly selected from each group, and the surface area of these cells was measured with NIH ImageJ software (version 1.52a).

### 4.7. Luciferase Reporter Activity

Forty-eight hours after plating cardiomyocytes in 6-well plate, culture medium was replaced with serum-free Dulbecco’s modified Eagle’s medium (DMEM). Then 1.4 µg pANF-luc or pET-1-luc plasmids were co-transfected with 14 ng pRL-SV40, which contains sea pansy Luc cDNA driven by a simian virus 40 promoter using Lipofectamin LTX and PLUS Reagent (Invitrogen). After a 2-h incubation with DNA-Lipofectamine complex, the cells were washed twice with serum-free DMEM and then treated with 1 µM sarpogrelate. Two hours after the treatment, the cells were stimulated with phenylephrine and further incubated for 48 h. Cell lysate preparation and promoter activity measurement were carried out as described previously [59,60].

### 4.8. Western Blotting

Total protein and nuclear fraction were extracted from cardiomyocytes and heart tissue and Western blotting was performed as previously described [52,55]. Ten µg of each fraction were resolved by SDS-PAGE. For Western blotting, anti-phospho-ERK1/2 (T202/Y204) antibody (Cell Signaling Technology, Danvers, MA, USA), anti-ERK1/2 antibody (Cell Signaling Technology), anti-phospho-GATA4 (S105) antibody (Abcam, Cambridge, United Kingdom), and anti-GATA4 antibody (Cell Signaling Technology) were used as primary antibodies, and anti-mouse antibody (MBL, Aichi, Japan) and anti-rabbit antibody (MBL) were used as secondary antibodies. Western blotting signals were visualized with an Amersham Imager 680 blot imager (GE Healthcare, Chicago, IL, USA) and quantified with NHI ImageJ software (version 1.52a).

### 4.9. ChIP Assay

A ChIP assay was performed as described previously [52,57]. In brief, cardiomyocytes were treated with formaldehyde to crosslink the DNA-protein complex. Nuclear fractions were extracted from the cells with nuclear lysis buffer (50 mM Tris-HCl, 140 mM NaCl, 10 mM EDTA, 1% SDS, 1% Triton X100) and sonicated to generate DNA fragmentation. After that, the target DNA-protein complex was immunoprecipitated with anti-GATA4 antibody (Santa Cruz Biotechnology, Dallas, TX, USA), and the immunocomplexes were captured by adding protein G beads (Santa Cruz Biotechnology). DNA was purified with a phenol-chloroform extraction and precipitated with ethanol. After the ChIP assay, quantitative real-time PCR was performed. Goat IgG (Santa Cruz Biotechnology) was used as a negative control for the assay.

### 4.10. Transverse Aortic Constriction and Drug Treatment

TAC surgery was performed as described previously [54,57]. In brief, C57BL/6J male mice (8 weeks old) were anesthetized with 1.0–1.5% isoflurane. The pleura was incised to the second rib, and the aortic arch was ligated using a 7-0 nylon suture ligature with a 27-gauge needle. One day after the surgery, the mice were randomly assigned to three groups: vehicle (Milli-Q water), 1 mg/kg sarpogrelate, and 5 mg/kg sarpogrelate. Sarpogrelate was dissolved with Milli-Q water and administrated orally by gastric gavage once a day for 8 weeks.

### 4.11. Echocardiography

Echocardiography was performed at 8 weeks after surgery as described previously [61,62]. In short, mice were anesthetized with 1.0–1.5% isoflurane, and two-dimensional (2D) images of the left ventricle and cardiac function were obtained with a 10–12 MHz probe and a Sonos 5500 Ultrasound System (Philips, Amsterdam, The Netherlands). Interventricular septum thickness at end-diastole (IVSd), left ventricular internal diameter end-diastole (LVIDd), left ventricular internal diameter end-systole (LVIDs), and left ventricular posterior wall thickness (LVPWT) were obtained from M-mode recordings. FS and LV mass were calculated as (LVIDd − LVIDs)/LVIDd × 100 (%) and 1.055 [(IVSd + LVIDd + LVPWT)^3^ − (LVIDd)^3^], respectively. LVMI is represented as the ratio of LV mass to tibia length.

### 4.12. Histological Analysis

FITC-conjugated WGA and MT staining were performed as described previously [57,62]. WGA-stained slices were photographed with a fluorescence microscope (LSM 510 META, Zeiss, Oberkochen, Germany), and the surface areas of 50 cardiomyocytes were measured with NIH ImageJ software (version 1.52a). The blood vessels in the MT-stained slices were photographed and perivascular fibrosis area was quantified using Adobe Photoshop Elements 2018 and NIH ImageJ software (version 1.52a).

### 4.13. Real-Time PCR

Real-time PCR was performed as previously described [57,63]. Rat ANF (forward, 5′- ATCACCAAGGGCTTCTTCCT -3′; reverse, 5′- CCTCATCTTCTACCGGCATC -3′) and rat BNP (forward, 5′-TTCCGGATCCAGGAGAGACTT-3′; reverse, 5′-CCTAAAACAACCTCAGCCCGT-3′) were used as primers of the hypertrophic response genes. The DNA levels of 5-HT_2A_ receptor and TPH1 and TPH2were quantified using the following primers: rat 5-HT_2A_ receptor (forward, 5′-AACGGTCCATCCACAGAG-3′; reverse, 5′-AACAGGAAGAACACGATGC-3′), rat TPH1 (forward, 5′-AGCATAACCAGCGCCATGAA-3′; reverse, 5′-GGCATCATTGACGACATCGAG-3′), and rat TPH2 (forward, 5′-GCCATGACACAGAAAGTTGTTG-3′; reverse, 5′- -3′). As internal controls, rat 18S (forward, 5′-CTTAGAGGGACAAGGGCG-3′; reverse, 5′-GGACATCTAAGGGCATCACA-3′) and rat GAPDH (forward, 5′-TTGCCATCAACGACCCCTTC-3′; reverse, 5′-TTGTCATGGATGACCTTGGG-3′) were used. The ANF, BNP, and 18S primers have sequence homologies between rat and mice.

### 4.14. Statistics

Parameters are shown as mean ± SEM. One-way analysis of variance (1-way ANOVA) and a Tukey–Kramer test were applied to determine the significance of the results. In addition, a Student independent *t*-test was conducted to compare results between two groups (control vs PE). A *p* value of < 0.05 was considered statistically significant.

## 5. Conclusions

This study suggests that sarpogrelate suppresses cardiomyocyte hypertrophy by inhibiting the ERK1/2–GATA4 signaling pathway, and that, in a mouse model of heart failure, sarpogrelate prevents the development of heart failure in both a 5-HT_2A_ receptor-dependent and independent manner.

## Figures and Tables

**Table 1:** Echocardiographic parameters of sham and TAC mice.

	Sham			TAC								
	Vehicle			Vehicle			SA 1 mg/kg			SA 5 mg/kg		
**LVPWd (mm)**	1.50	±	0.07	2.05	±	0.09 **	1.83	±	0.14	1.55	±	0.08 ^††^
**LVIDd (mm)**	2.67	±	0.12	2.94	±	0.16	2.75	±	0.12	2.79	±	0.13
**FS (%)**	57.4	±	2.2	37.7	±	2.4 **	41.9	±	3.7 **	50.7	±	0.8 ^††^
**R-R int (sec)**	0.101	±	0.001	0.103	±	0.001	0.101	±	0.001	0.102	±	0.002
**LVMI (mg/mm)**	7.0	±	0.2	13.0	±	0.8 **	10.8	±	1.0 **	9.5	±	1.2 ^†^
**HW (mg)**	118.3	±	2.2	192.1	±	12.7 **	166.6	±	2.0 **	151.0	±	3.6 ^†^
**TL (mm)**	22.5	±	0.6	22.5	±	0.4	22.1	±	0.7	23.2	±	0.6
**HW/TL (mg/mm)**	5.3	±	0.2	8.6	±	0.6 **	7.6	±	0.2 **	6.5	±	0.2 ^†^

** *p* < 0.01 vs. Sham + vehicle group. † *p* < 0.05, †† *p* < 0.01 vs. TAC + vehicle group. The values shown are the mean ± SEM for 5–8 mice from each of the sham and TAC groups. Abbreviations: LVPWd, left ventricle posterior wall dimensions; LVIDd, left ventricular internal diameter end-diastole; FS, fractional shortening; R-R int: R-R interval; LVMI, left ventricular mass index; HW: heart weight; TL: tibia length; HW/TL: heart weight to tibia length ratio.

## Supplementary Materials

The following are available online at https://www.mdpi.com/article/10.3390/ph14121268/s1, Figure S1: Knockdown of the 5-HT_2A_ receptor did not affect the anti-hypertrophy effect of sarpogrelate on PE-induced cardiomyocyte hypertrophy.

## References

[1] Bragazzi N.L., Zhong W., Shu J., Abu Much A., Lotan D., Grupper A., Younis A., Dai H. (2021). Burden of Heart Failure and Underlying Causes in 195 Countries and Territories from 1990 to 2017. Eur. J. Prev. Cardiol..

[2] Ziaeian B., Fonarow G.C. (2016). Epidemiology and Aetiology of Heart Failure. Nat. Rev. Cardiol..

[3] NCD Countdown 2030 Collaborators (2018). NCD Countdown 2030: Worldwide Trends in Non-Communicable Disease Mortality and Progress Towards Sustainable Development Goal Target 3.4. Lancet.

[4] Callender T., Woodward M., Roth G., Farzadfar F., Lemarie J.C., Gicquel S., Atherton J., Rahimzadeh S., Ghaziani M., Shaikh M. (2014). Heart Failure Care in Low- and Middle-Income Countries: A Systematic Review and Meta-Analysis. PLoS Med..

[5] Flather M.D., Yusuf S., Køber L., Pfeffer M., Hall A., Murray G., Torp-Pedersen C., Ball S., Pogue J., Moyé L. (2000). Long-term ACE-Inhibitor Therapy in Patients with Heart Failure or Left-Ventricular Dysfunction: A Systematic Overview of Data from Individual Patients. Lancet.

[6] Metra M., Teerlink J.R. (2017). Heart Failure. Lancet.

[7] Elvira K.S. (2021). Microfluidic Technologies for Drug Discovery and Development: Friend or Foe?. Trends Pharmacol. Sci..

[8] Lotfi Shahreza M., Ghadiri N., Mousavi S.R., Varshosaz J., Green J.R. (2018). A Review of Network-Based Approaches to Drug Repositioning. Brief Bioinform..

[9] Panchapakesan U., Pollock C. (2018). Drug Repurposing in Kidney Disease. Kidney Int..

[10] Yella J.K., Yaddanapudi S., Wang Y., Jegga A.G. (2018). Changing Trends in Computational Drug Repositioning. Pharmaceuticals.

[11] Jin G., Wong S.T.C. (2014). Toward Better Drug Repositioning: Prioritizing and Integrating Existing Methods into Efficient Pipelines. Drug Discov. Today.

[12] Shinohara Y., Nishimaru K., Sawada T., Terashi A., Handa S., Hirai S., Hayashi K., Tohgi H., Fukuuchi Y., Uchiyama S. (2008). Sarpogrelate-Aspirin Comparative Clinical Study for Efficacy and Safety in Secondary Prevention of Cerebral Infarction (S-ACCESS): A Randomized, Double-Blind, Aspirin-Controlled Trial. Stroke.

[13] Shinohara Y., Nishimaru K. (2009). Sarpogrelate versus Aspirin in Secondary Prevention of Cerebral Infarction: Differential Efficacy in Diabetes?: Subgroup Analysis from S-Access. Stroke.

[14] Lee H., Chae S., Park J., Bae J., Go E.B., Kim S.J., Kim H., Hwang D., Lee S.W., Lee S.Y. (2016). Comprehensive Proteome Profiling of Platelet Identified a Protein Profile Predictive of Responses to an Antiplatelet Agent Sarpogrelate. Mol. Cell Proteom..

[15] Kim D.H., Choi B.H., Ku S.K., Park J.H., Oh E., Kwak M.K.K. (2016). Beneficial Effects of Sarpogrelate and Rosuvastatin in High Fat Diet/Streptozotocin-Induced Nephropathy in Mice. PLoS ONE..

[16] Ku C.A., Ryals R.C., Jiang D., Coyner A.S., Weller K.K., Sinha W., Robb B.M., Yang P., Pennesi M.E. (2018). The Role of ERK1/2 Activation in Sarpogrelate-Mediated Neuroprotection. Invest. Ophthalmol. Vis. Sci..

[17] Su Y., Mao N., Li M., Dong X., Lin F.Z., Xu Y., Li Y.B. (2013). Sarpogrelate Inhibits the Expression of ICAM-1 and Monocyte-Endothelial Adhesion Induced by High Glucose in Human Endothelial Cells. Mol. Cell Biochem..

[18] Sun Y.M., Su Y., Jin H.B., Li J., Bi S. (2011). Sarpogrelate Protects against High Glucose-Induced Endothelial Dysfunction and Oxidative Stress. Int. J. Cardiol..

[19] Ikeda K., Tojo K., Otsubo C., Udagawa T., Kumazawa K., Ishikawa M., Tokudome G., Hosoya T., Tajima N., Claycomb W.C. (2005). 5-Hydroxytryptamine Synthesis in Hl-1 Cells and Neonatal Rat Cardiocytes. Biochem. Biophys. Res. Commun..

[20] Hara K., Hirowatari Y., Yoshika M., Komiyama Y., Tsuka Y., Takahashi H. (2004). The Ratio of Plasma to Whole-Blood Serotonin may Be a Novel Marker of Atherosclerotic Cardiovascular Disease. J. Lab. Clin. Med..

[21] Mawe G.M., Hoffman J.M. (2013). Serotonin Signalling in the Gut-Functions, Dysfunctions and Therapeutic Targets. Nat. Rev. Gastroenterol. Hepatol..

[22] Villeneuve C., Caudrillier A., Ordener C., Pizzinat N., Parini A., Mialet-Perez J. (2009). Dose-Dependent Activation of Distinct Hypertrophic Pathways by Serotonin in Cardiac Cells. Am. J. Physiol. Heart Circ. Physiol..

[23] Mialet-Perez J., D’Angelo R., Villeneuve C., Ordener C., Nègre-Salvayre A., Parini A., Vindis C. (2012). Serotonin 5-HT2A Receptor-Mediated Hypertrophy is Negatively Regulated by Caveolin-3 in Cardiomyoblasts and Neonatal Cardiomyocytes. J. Mol. Cell Cardiol..

[24] Park-Windhol C., Zhang P., Zhu M., Su J., Chaves L., Maldonado A.E., King M.E., Rickey L., Cullen D., Mende U. (2012). Gq/11-Mediated Signaling and Hypertrophy in Mice with Cardiac-Specific Transgenic Expression of Regulator of G-Protein Signaling 2. PLoS ONE.

[25] Gutkind J.S., Offermanns S. (2009). A New Gq-Initiated MAPK Signaling Pathway in the Heart. Dev. Cell.

[26] Bueno O.F., Molkentin J.D. (2002). Involvement of Extracellular Signal-Regulated Kinases 1/2 in Cardiac Hypertrophy and Cell Death. Circ. Res..

[27] Streicher J.M., Ren S., Herschman H., Wang Y. (2010). MAPK-Activated Protein Kinase-2 in Cardiac Hypertrophy and Cyclooxygenase-2 Regulation in Heart. Circ. Res..

[28] Wei Z., Liu H.T. (2002). MAPK Signal Pathways in the Regulation of Cell Proliferation in Mammalian Cells. Cell Res..

[29] Jagodzik P., Tajdel-Zielinska M., Ciesla A., Marczak M., Ludwikow A. (2018). Mitogen-Activated Protein Kinase Cascades in Plant Hormone Signaling. Front. Plant. Sci..

[30] Purcell N.H., Wilkins B.J., York A., Saba-El-Leil M.K., Meloche S., Robbins J., Molkentin J.D. (2007). Genetic Inhibition of Cardiac ERK1/2 Promotes Stress-Induced Apoptosis and Heart Failure but Has No Effect on Hypertrophy In Vivo. Proc. Natl. Acad. Sci. USA.

[31] Mutlak M., Kehat I. (2015). Extracellular Signal-Regulated Kinases 1/2 as Regulators of Cardiac Hypertrophy. Front. Pharmacol..

[32] Song J., Xie Q., Wang L., Lu Y., Liu P., Yang P., Chen R., Shao C., Qiao C., Wang Z. (2019). The TIR/BB-Loop Mimetic AS-1 Prevents Ang Ii-Induced Hypertensive Cardiac Hypertrophy via NF-κB Dependent Downregulation of miRNA-143. Sci. Rep..

[33] Markou T., Lazou A. (2002). Phosphorylation and Activation of Mitogen- and Stress-Activated Protein Kinase-1 in Adult Rat Cardiac Myocytes by G-Protein-Coupled Receptor Agonists Requires Both Extracellular-Signal-Regulated Kinase and p38 Mitogen-Activated Protein Kinase. Biochem. J..

[34] Mutlak M., Kehat I. (2021). Dual Specific Phosphatases (DUSPs) in Cardiac Hypertrophy and Failure. Cell Signal..

[35] Sanna B., Bueno O.F., Dai Y.-S., Wilkins B.J., Molkentin J.D. (2005). Direct and Indirect Interactions between Calcineurin-NFAT and MEK1-Extracellular Signal-Regulated Kinase 1/2 Signaling Pathways Regulate Cardiac Gene Expression and Cellular Growth. Mol. Cell Biol..

[36] Van Rooij E., Doevendans P.A., De Theije C.C., Babiker F.A., Molkentin J.D., De Windt L.J. (2002). Requirement of Nuclear Factor of Activated T-Cells in Calcineurin-Mediated Cardiomyocyte Hypertrophy. J. Biol. Chem..

[37] Morimoto T., Hasegawa K., Wada H., Kakita T., Kaburagi S., Yanazume T., Sasayama S. (2001). Calcineurin-GATA4 Pathway Is Involved in Beta-Adrenergic Agonist-Responsive Endothelin-1 Transcription in Cardiac Myocytes. J. Biol. Chem..

[38] Cohn J.N., Ferrari R., Sharpe N. (2000). Cardiac Remodeling-Concepts and Clinical Implications: A Consensus Paper from an International Forum on Cardiac Remodeling. J. Am. Coll. Cardiol..

[39] Archer C.R., Robinson E.L., Drawnel F.M., Roderick H.L. (2017). Endothelin-1 Promotes Hypertrophic Remodelling of Cardiac Myocytes by Activating Sustained Signalling and Transcription Downstream of Endothelin Type A Receptors. Cell Signal..

[40] Dorn G.W., Force T. (2005). Protein Kinase cascades in the Regulation of Cardiac Hypertrophy. J. Clin. Invest..

[41] Selim A.M., Sarswat N., Kelesidis I., Iqbal M., Chandra R., Zolty R. (2017). Plasma Serotonin in Heart Failure: Possible Marker and Potential Treatment Target. Heart Lung Circ..

[42] Lairez O., Cognet T., Schaak S., Calise D., Guilbeau-Frugier C., Parini A., Mialet-Perez J. (2013). Role of Serotonin 5-HT2A Receptors in the Development of Cardiac Hypertrophy in Response to Aortic Constriction in Mice. J. Neural. Transm..

[43] Guillet-Deniau I., Burnol A.F., Girard J. (1997). Identification and Localization of a Skeletal Muscle Secrotonin 5-HT2A Receptor Coupled to the Jak/STAT Pathway. J. Biol. Chem..

[44] Hall C., Gehmlich K., Denning C., Pavlovic D. (2021). Complex Relationship between Cardiac Fibroblasts and Cardiomyocytes in Health and Disease. J. Am. Heart Assoc..

[45] Si L., Xu J., Yi C., Xu X., Wang F., Gu W., Zhang Y., Wang X. (2014). Asiatic Acid Attenuates Cardiac Hypertrophy by Blocking Transforming Growth Factor-β1-Mediated Hypertrophic Signaling In Vitro and In Vivo. Int. J. Mol. Med..

[46] Kehat I., Davis J., Tiburcy M., Accornero F., Saba-El-Leil M.K., Maillet M., York A.J., Lorenz J.N., Zimmermann W.H., Meloche S. (2011). Extracellular Signal-Regulated Kinases 1 and 2 Regulate the Balance between Eccentric and Concentric Cardiac growth. Circ. Res..

[47] Bueno O.F., De Windt L.J., Tymitz K.M., Witt S.A., Kimball T.R., Klevitsky R., Hewett T.E., Jones S.P., Lefer D.J., Peng C.F. (2000). The MEK1-ERK1/2 Signaling Pathway Promotes Compensated Cardiac Hypertrophy in Transgenic Mice. EMBO J..

[48] Chung I., Lip G.Y.H. (2006). Platelets and Heart Failure. Eur. Heart J..

[49] Tanaka-Totoribe N., Hidaka M., Gamoh S., Yokota A., Nakamura E., Kuwabara M., Tsunezumi J., Yamamoto R. (2020). Effects of M-1, a Major Metabolite of Sarpogrelate, on 5-HT-Induced Constriction of Isolated Human Internal Thoracic Artery. Biol. Pharm. Bull..

[50] Sanganalmath S.K., Babick A.P., Barta J., Kumamoto H., Takeda N., Dhalla N.S. (2008). Antiplatelet Therapy Attenuates Subcellular Remodelling in Congestive Heart Failure. J. Cell Mol. Med..

[51] Choi J.H., Cho J.R., Park S.M., Shaha K.B., Pierres F., Sumiya T., Chun K.J., Kang M.K., Choi S., Lee N. (2017). Sarpogrelate Based Triple Antiplatelet Therapy Improved Left Ventricular Systolic Function in Acute Myocardial Infarction: Retrospective Study. Yonsei Med. J..

[52] Funamoto M., Sunagawa Y., Katanasaka Y., Shimizu K., Miyazaki Y., Sari N., Shimizu S., Mori K., Wada H., Hasegawa K. (2021). Histone acetylation Domains are Differentially Induced during Development of Heart Failure in Dahl Salt-Sensitive Rats. Int. J. Mol. Sci..

[53] Morimoto T., Sunagawa Y., Kawamura T., Takaya T., Wada H., Nagasawa A., Komeda M., Fujita M., Shimatsu A., Kita T. (2008). The Dietary Compound Curcumin Inhibits p300 Histone Acetyltransferase Activity and Prevents Heart Failure in Rats. J. Clin. Invest..

[54] Sari N., Katanasaka Y., Honda H., Miyazaki Y., Sunagawa Y., Funamoto M., Shimizu K., Shimizu S., Wada H., Hasegawa K. (2020). Cacao Bean Polyphenols Inhibit Cardiac Hypertrophy and Systolic Dysfunction in Pressure Overload-induced Heart Failure Model Mice. Planta Med..

[55] Morimoto T., Hasegawa K., Kaburagi S., Kakita T., Wada H., Yanazume T., Sasayama S. (2000). Phosphorylation of GATA-4 Is Involved in α1-Adrenergic Agonist-Responsive Transcription of the Endothelin-1 Gene in Cardiac Myocytes. J. Biol. Chem..

[56] Dignam J.D., Lebovitz R.M., Roeder R.G. (1983). Accurate Transcription Initiation by RNA Polymerase II in a Soluble Extract from Isolated Mammalian Nuclei. Nucl. Acid. Res..

[57] Shimizu K., Sunagawa Y., Funamoto M., Wakabayashi H., Genpei M., Miyazaki Y., Katanasaka Y., Sari N., Shimizu S., Katayama A. (2020). The Synthetic Curcumin Analogue GO-Y030 Effectively Suppresses the Development of Pressure Overload-induced Heart Failure in Mice. Sci. Rep..

[58] Sunagawa Y., Shimizu K., Katayama A., Funamoto M., Shimizu K., Nurmila S., Shimizu S., Miyazaki Y., Katanasaka Y., Hasegawa K. (2021). Metformin Suppresses Phenylephrine-Induced Hypertrophic Responses by Inhibiting p300-HAT Activity in Cardiomyocytes. J. Pharmacol. Sci..

[59] Sunagawa Y., Morimoto T., Takaya T., Kaichi S., Wada H., Kawamura T., Fujita M., Shimatsu A., Kita T., Hasegawa K. (2010). Cyclin-Dependent Kinase-9 Is a Component of the p300/GATA4 Complex Required for Phenylephrine-Induced Hypertrophy in Cardiomyocytes. J. Biol. Chem..

[60] Suzuki H., Katanasaka Y., Sunagawa Y., Miyazaki Y., Funamoto M., Wada H., Hasegawa K., Morimoto T. (2016). Tyrosine Phosphorylation of RACK1 Triggers Cardiomyocyte Hypertrophy by Regulating the Interaction between p300 and GATA4. Biochim. Biophys. Acta.

[61] Sunagawa Y., Funamoto M., Shimizu K., Shimizu S., Sari N., Katanasaka Y., Miyazaki Y., Kakeya H., Hasegawa K., Morimoto T. (2021). Curcumin, an Inhibitor of p300-HAT Activity, Suppresses the Development of Hypertension-Induced Left Ventricular Hypertrophy with Preserved Ejection Fraction in Dahl Rats. Nutrients.

[62] Sunagawa Y., Sono S., Katanasaka Y., Funamoto M., Hirano S., Miyazaki Y., Hojo Y., Suzuki H., Morimoto E., Marui A. (2014). Optimal Dose-Setting Study of Curcumin for Improvement of Left Ventricular Systolic Function after Myocardial Infarction in Rats. J. Pharmacol. Sci..

[63] Sunagawa Y., Funamoto M., Sono S., Shimizu K., Shimizu S., Genpei M., Miyazaki Y., Katanasaka Y., Morimoto E., Ueno M. (2018). Curcumin and Its Demethoxy Derivatives Possess p300 HAT Inhibitory Activity and Suppress Hypertrophic Responses in Cardiomyocytes. J. Pharmacol. Sci..

