# Supplementary material for: The Selective Serotonin 2A Receptor Antagonist Sarpogrelate Prevents Cardiac Hypertrophy and Systolic Dysfunction via Inhibition of the ERK1/2–GATA4 Signaling Pathway
Source: Pharmaceuticals (Basel). 2021 Dec 5;14(12):1268. doi: 10.3390/ph14121268 (PMC8708651; doi:10.3390/ph14121268)
Supplement: Supplementary file 1 [file pharmaceuticals-14-01268-s001.zip › pharmaceuticals-1387453-supplementary.pdf]

Supplementary materials.

1.1. Small interfering RNA transfection of 5-HT_{2A} receptor

Small interfering RNA (siRNA) transfection was performed as described previously [1,2]. si-5-HT_{2A} receptors (Rn_Htr2a_4685, Sigma-Aldrich) were transfected to cardiomyocytes with lipofectamine RNAiMAX (Invitrogen) in accordance with the manufacturer's instructions. Twelve hours after transfection, the cells were pretreated with sarpogrelate for 2 hours and then stimulated with PE for 48 hours in serum-free medium. Mission® siRNA Universal Negative Control #1 (Sigma-Aldrich) was used as a negative control for siRNA transfections.

1.2. Statistics

Values are shown as mean ± SEM. The Tukey-Kramer test was used to determine significant differences. A p value of < 0.05 was considered statistically significant.

References

1. Funamoto, M.; Sunagawa, Y.; Katanasaka, Y.; Shimizu, K.; Miyazaki, Y.; Sari, N.; Shimizu, S.; Mori, K.; Wada, H.; Hasegawa, K.; et al. Histone acetylation domains are differentially induced during development of heart failure in dahl salt-sensitive rats. *Int J Mol Sci.* **2021**, *22*, 1771, doi:10.3390/ijms22041771.
2. Suzuki, H.; Katanasaka, Y.; Sunagawa, Y.; Miyazaki, Y.; Funamoto, M.; Wada, H.; Hasegawa, K.; Morimoto, T. Tyrosine phosphorylation of RACK1 triggers cardiomyocyte hypertrophy by regulating the interaction between p300 and GATA4. *Biochim Biophys Acta.* **2016**, *1862*, 1544–1557,

doi:10.1016/j.bbadis.2016.05.006.

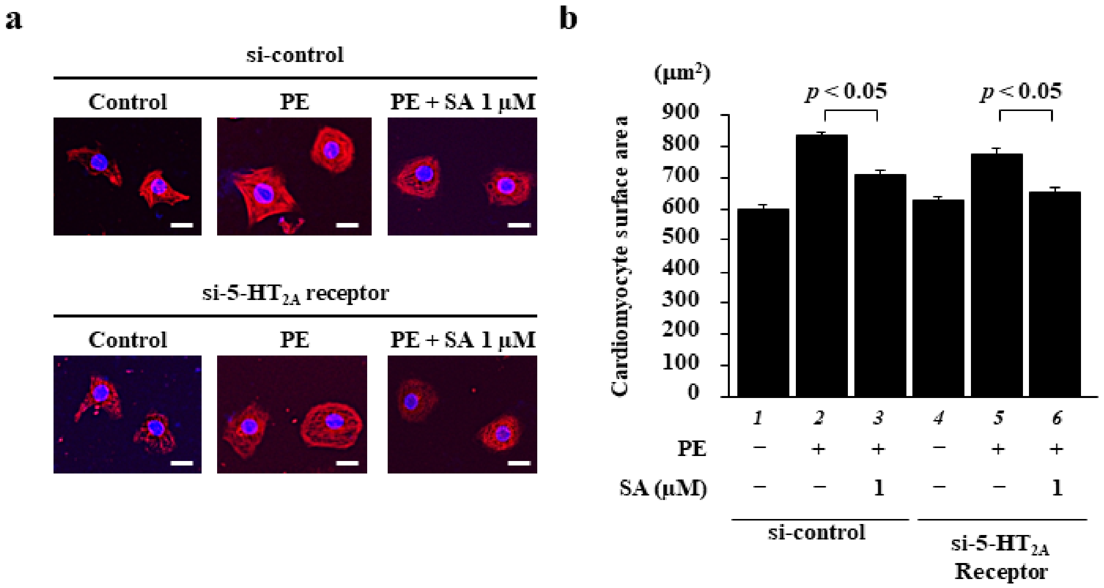

Supplementary Figure S1 Knockdown of the 5-HT2A receptor did not affect the anti-hypertrophy effect of sarpogrelate on PE-induced cardiomyocyte hypertrophy. **(a)** Cardiomyocytes were transfected with si-5-HT2A receptor siRNA. Immunofluorescence staining was performed using anti-MHC antibody and Alexa Fluor 555-conjuagted anti-mouse IgG. Scale bar: 20 μm **(b)** Measurement of the surface area of these cells was performed using ImageJ software (version 1.52a). The values are presented as mean ± SEM of 3 individual experiments.
